# Enhancing the support of China's long-term care insurance system for parents of the only child

**DOI:** 10.3389/fpubh.2025.1641398

**Published:** 2025-10-06

**Authors:** Weidong Wu, Ruizi Zhou

**Affiliations:** ^1^School of Humanities, Jinan University, Zhuhai, China; ^2^Youth League Committee, Jinan University, Guangzhou, China

**Keywords:** long-term care insurance system, parents of the only child, China, older adult, aging

## Introduction

With population aging accelerating, China is improving its medical security system and healthcare equity to meet older adults' care needs. The Fifth Sample Survey indicates there were about 35 million disabled older adults in 2021, projected to reach 46 million by 2035 and 58 million by 2050 (1). This demographic shift highlights the urgent need for innovative eldercare solutions. Long-term care insurance (LTCI), introduced in China in 2016, addresses care costs and reduces family caregiving burdens. By 2024, the LTCI system had expanded to 49 pilot cities, exceeding previous social insurance programs in longevity, coverage, and population served (2). As of the end of 2024, a total of 187.86 million people participated in long-term care insurance across 49 pilot cities, with 1.46 million individuals receiving benefits (3). This broad implementation highlights the government's commitment to addressing older adult care, yet also reveals areas that require further refinement.

One such area involves support for parents of only children, a group with unique and growing vulnerabilities. This issue is rooted in China's family planning policy, which, since the 1970s, aimed to harmonize population growth with economic development by limiting most families to a single child. Over more than three decades of this policy's enforcement, a distinct demographic has emerged: only-child families (4). In 2020, parents of only children totaled 400 million, representing 28.5% of China's population (5). From 2023 to 2032, most first-generation parents of only children will be aged 75–84, surpassing the “disability threshold” and facing a marked decline in self-care ability and increased need for caregiving (6). Compared with those in multi-child families, older adult parents in only-child households often face a greater risk of lacking both caregivers and sufficient funds for care (7).

Focusing on these challenges, Article 31 of the Population and Family Planning Law of the People's Republic of China states that during the period when the state advocated for one child per couple, those eligible for rewards and assistance as older adults from family planning families shall continue to receive relevant benefits, as well as necessary priority and care in elder welfare and services. Within this framework, long-term care insurance plays a vital role by offering targeted support to only-child families, further easing their caregiving burden and promoting greater equity in eldercare services across China.

## Expanding coverage for parents of the only child

Currently, China's LTCI system remains in the pilot phase and is limited to select areas. Elders outside these regions are excluded, and many within pilot cities also lack coverage. In most cases, LTCI primarily supports urban employees with social insurance, offering insufficient coverage for urban and rural residents (8). In the 49 pilot cities, the coverage rate of long-term care insurance participants was still below 55% of the total population in 2023 (2). A 2015 survey across five provinces and cities found that urban first-generation only-child parents mainly rely on self-care, with low levels of family-based eldercare support (9). Given their unique challenges, future LTCI pilots should prioritize including these parents to strengthen institutional support for this vulnerable group. Both theory and practice in statistics have shown that a larger pool of insured individuals leads to greater efficiency in insurance systems (10). Higher enrollment also enhances risk sharing in long-term care insurance.

Focusing on pilot cities where LTCI coverage has not yet been expanded to all urban and rural residents, future policy directions could prioritize institutional support for parents who have lost their only child and parents of only children who have become disabled due to accidental injuries. Based on estimates from China's seventh national census and previous data, there were about 2.888 million mothers aged 50 and above who had lost their only child by 2020 (11). These parents, due to the death or disability of their only child, experience generational cleavage and lack family care in old age. In addition to age-related vulnerability, they also face external family vulnerability, which is linked to past national policies. According to vulnerability theory, the state should implement welfare policies to address these individuals' vulnerabilities (12).

Policy support for these families is grounded in relevant legislation. Article 32 of the Population and Family Planning Law of the People's Republic of China stipulates that couples holding the “Certificate of Honor for Parents of the Only Child” and whose only child has become disabled or died in an accident are entitled to receive assistance according to regulations. As the LTCI system continues to expand across the nation, it is vital to maintain necessary prioritization for parents of the only child.

## Strengthening insurance enrollment support for parents of the only child

Preferential measures should moderately favor parents of only children, such as by increasing government subsidies to ease their financial burden when joining the insurance program. Many pilot LTCI cities already provide participant subsidies through various financing models. By raising subsidy standards specifically for only-child parents, more targeted support can be offered to this group. According to a survey conducted in Nanjing, 67.3% of older adult residents are unwilling to participate in LTCI (13). This relatively low participation rate among older adults, including parents of the only child, highlights the need for further action.

In addition to Article 31, Article 33 of the Population and Family Planning Law of the People's Republic of China stipulates that “local governments at all levels shall give priority to poor families implementing family planning in terms of poverty alleviation loans, work-for-relief programs, poverty alleviation projects, and social assistance.” By appropriately increasing local government financial subsidies, it may be possible to boost the enthusiasm of parents of the only child to participate in LTCI.

## Enriching the benefits provided to parents of the only child

To better support parents of the only child, the scope of benefits under LTCI should be appropriately expanded. This expansion should include not only increased economic compensation and a higher reimbursement ratio for medical and care expenses, but also a gradual inclusion of home-based self-care costs within LTCI's payment coverage. From 2020 to 2024, the 49 pilot cities' long-term care insurance funds had a cumulative expenditure of 65.39 billion RMB and revenue of 118.53 billion RMB, resulting in a surplus of 53.14 billion RMB (2). This substantial surplus provides room for policy initiatives. Benefit provision standards should be flexibly and dynamically adjusted according to the economic status of parents of the only child. For those who are economically disadvantaged, it is especially important to establish more diverse and comprehensive benefit models that address their unique challenges.

In parallel, there is a pressing need to optimize the LTCI service catalog system so that it can offer a broader range of differentiated services tailored to parents of the only child who require care. Services such as daily living assistance, rehabilitation training, and medical care should be provided in ways that address the specific needs of this population. Comparative analysis of the 2018 Chinese Longitudinal Healthy Longevity Survey shows that older adult parents with only one child have fewer caregivers, and each caregiver bears a heavier burden than those in multi-child families (14). Heavier caregiving burdens may increase the risk of intergenerational contract failure between parents and children. According to intergenerational contract theory, this informal agreement often places parents in a weaker position, requiring government policy support to protect their wellbeing when the contract fails (15).

However, the current care system in China is hindered by persistent shortcomings, such as a shortage of care institutions and qualified professional caregivers. In 2024, China had 8,837 long-term care institutions with 292,800 care workers, almost 40,000 fewer than in 2021 (2). Care worker shortages persist alongside low pay, poor recognition, and over 30% annual turnover (1). Japan has faced similar issues since 2000, addressing them by improving pay and introducing care robots and monitoring devices (16).

## Conclusion

The older adult parents of the only child in China face relatively higher risks in later life, which is directly related to the country's implementation of the one-child policy during a specific historical period. As such, it is necessary for China to assume compensatory responsibilities for this group. Policies for older adult care and security should be formulated specifically to address the unique needs of parents of the only child, and these policies should be effectively embodied in relevant programs within the older adult security system (4). Currently, the LTCI system in China does not provide sufficient preferential treatment for parents of the only child and lacks full consideration of their particular circumstances. At the stage of severe disability, parents of the only child are less likely to receive care from their children, which makes the support of the LTCI system especially critical. The LTCI scheme should be designed to offer preferential policies for these parents in terms of premium base, benefit payments, and benefit types.
